# Implementing Pharmacogenetic Testing in Gastrointestinal Cancers (IMPACT-GI): Study Protocol for a Pragmatic Implementation Trial for Establishing *DPYD* and *UGT1A1* Screening to Guide Chemotherapy Dosing

**DOI:** 10.3389/fonc.2022.859846

**Published:** 2022-07-05

**Authors:** Lisa A. Varughese, Madhuri Bhupathiraju, Glenda Hoffecker, Shannon Terek, Margaret Harr, Hakon Hakonarson, Christine Cambareri, Jessica Marini, Jeffrey Landgraf, Jinbo Chen, Genevieve Kanter, Kelsey S. Lau-Min, Ryan C. Massa, Nevena Damjanov, Nandi J. Reddy, Randall A. Oyer, Ursina R. Teitelbaum, Sony Tuteja

**Affiliations:** ^1^Division of Translational Medicine and Human Genetics, Department of Medicine, Perelman School of Medicine, University of Pennsylvania, Philadelphia, PA, United States; ^2^Center for Applied Genomics, The Children’s Hospital of Philadelphia, Philadelphia, PA, United States; ^3^Department of Pharmacy, Hospital of the University of Pennsylvania, Philadelphia, PA, United States; ^4^Information Services Applications, Penn Medicine, University of Pennsylvania, Philadelphia, PA, United States; ^5^Department of Biostatistics, Epidemiology and Informatics, Perelman School of Medicine, University of Pennsylvania, Philadelphia, PA, United States; ^6^Division of Medical Ethics and Health Policy, Department of Medicine, Perelman School of Medicine, University of Pennsylvania, Philadelphia, PA, United States; ^7^Division of Hematology/Oncology, Department of Medicine, Perelman School of Medicine, University of Pennsylvania, Philadelphia, PA, United States; ^8^Ann B. Barshinger Cancer Institute, Lancaster General Health, Penn Medicine, Lancaster, PA, United States

**Keywords:** pharmacogenetics, cancer, DPYD, UGT1A1, chemotherapy, toxicity, pragmatic trial, implementation science

## Abstract

**Background:**

Fluoropyrimidines (fluorouracil [5-FU], capecitabine) and irinotecan are commonly prescribed chemotherapy agents for gastrointestinal (GI) malignancies. Pharmacogenetic (PGx) testing for germline *DPYD* and *UGT1A1* variants associated with reduced enzyme activity holds the potential to identify patients at high risk for severe chemotherapy-induced toxicity. Slow adoption of PGx testing in routine clinical care is due to implementation barriers, including long test turnaround times, lack of integration in the electronic health record (EHR), and ambiguity in test cost coverage. We sought to establish PGx testing in our health system following the Exploration, Preparation, Implementation, Sustainment (EPIS) framework as a guide. Our implementation study aims to address barriers to PGx testing.

**Methods:**

The Implementing Pharmacogenetic Testing in Gastrointestinal Cancers (IMPACT-GI) study is a non-randomized, pragmatic, open-label implementation study at three sites within a major academic health system. Eligible patients with a GI malignancy indicated for treatment with 5-FU, capecitabine, or irinotecan will undergo PGx testing prior to chemotherapy initiation. Specimens will be sent to an academic clinical laboratory followed by return of results in the EHR with appropriate clinical decision support for the care team. We hypothesize that the availability of a rapid turnaround PGx test with specific dosing recommendations will increase PGx test utilization to guide pharmacotherapy decisions and improve patient safety outcomes. Primary implementation endpoints are feasibility, fidelity, and penetrance. Exploratory analyses for clinical effectiveness of genotyping will include assessing grade ≥3 treatment-related toxicity using available clinical data, patient-reported outcomes, and quality of life measures.

**Conclusion:**

We describe the formative work conducted to prepare our health system for *DPYD* and *UGT1A1* testing. Our prospective implementation study will evaluate the clinical implementation of this testing program and create the infrastructure necessary to ensure sustainability of PGx testing in our health system. The results of this study may help other institutions interested in implementing PGx testing in oncology care.

**Clinical Trial Registration:**

https://clinicaltrials.gov/ct2/show/NCT04736472, identifier [NCT04736472].

## Introduction

Clinical pharmacogenetics (PGx) is a promising tool that harnesses an individual’s germline genetic information to optimize prescribing decisions and improve medication-related outcomes. Advances in genomic technologies and the increasing number of clinical practice guidelines have led to health system-wide precision medicine initiatives to support PGx programs (1–4). PGx testing can be leveraged in the oncology setting to guide chemotherapy dosing to minimize the severity of treatment-induced toxicity, thereby reducing the potential for costly emergency department visits and/or hospitalizations (5–7). Fluoropyrimidines (fluorouracil [5-FU] and its oral prodrug capecitabine) and irinotecan are well-known systemic chemotherapy agents used in a wide variety of tumors. Treatment-related toxicities manifesting as neutropenia, diarrhea, mucositis, and hand-foot syndrome are prevalent in 35-50% of patients receiving combination regimens (8). While interindividual differences in the severity of adverse events is partially due to clinical factors such as age, sex, organ dysfunction, and performance status, common genetic variation can further explain differences in chemotherapy response as it relates to its safety profile.

The *DPYD* gene encodes dihydropyrimidine dehydrogenase (DPD), the primary enzyme responsible for degrading more than 80% of an administered fluoropyrimidine dose. While fluoropyrimidines are generally well-tolerated, decreased DPD activity is associated with a greater than four-fold risk of severe or fatal toxicity from standard dosing (9). Further data suggests that carriers with variant alleles encoding for decreased DPD function have a 25.6-times increased risk of treatment-related death following standard dose fluoropyrimidine in solid tumors (10). Partial or complete DPD deficiency stems from approximately 40 different genetic aberrations, including exon skipping, deletions, frameshifts, missense mutations, and polymorphisms (11). The relationship between four *DPYD* variants (c.1905+1G>A [*2A], c.1679T>G [*13], c.2846A>T, and c.1129-5923 C>G/c.1236G>A [HapB3]) and fluoropyrimidine-induced toxicity have primarily been studied in populations of European ancestry, with a combined carrier frequency of approximately 2-8% (10, 12). Additional reports suggest the c.557A>G variant, evident in 3-5% in individuals of African ancestry, is also associated with reduced DPD activity and fluoropyrimidine toxicity (13, 14).

Results from prospective trials testing for common *DPYD* variants prior to treatment justify recommendations to perform initial dose reductions in variant carriers (5, 12). The Clinical Pharmacogenetics Implementation Consortium (CPIC) guidelines provide fluoropyrimidine dosing recommendations based on *DPYD* gene activity score, where a variant score of 1 corresponds to normal function, 0.5 as reduced function, and 0 as no function. Phenotypes, or metabolizer status, are assigned based on the sum of the two lowest variant activity scores. Individuals found to be intermediate metabolizers (Activity Score 1 or 1.5) are recommended to receive a 50% reduction in the starting dose. Poor metabolizers are recommended to avoid fluoropyrimidines (Activity Score 0) or a strongly reduced dose (<25% of the normal starting dose) due to the potential for life-threatening or fatal toxicity (Activity Score 0.5) (15).

Concurrent *UGT1A1* testing with *DPYD* screening can be considered to guide irinotecan dosing given its higher frequency of polymorphisms, regardless of if the agent is administered alongside a fluoropyrimidine or used in future lines of therapy. The UDP-glucuronosyltransferase 1A1 (UGT1A1) enzyme is encoded by *UGT1A1* and is responsible for inactivating SN-38 (the active metabolite of irinotecan) following glucuronidation. *UGT1A1* genotype assay results are reported by using the star (*) allele nomenclature or by the number of thymine-adenine (TA) repeats in the gene promoter region; wild-type contains six TA repeats [(TA)6TAA or 6/6]. Homozygosity or compound heterozygosity in the *28 [(TA)7TAA or 7/7] and *6 (c.211G>A) alleles are associated with reduced UGT1A1 activity, resulting in increased exposure to SN-38 and a higher risk of severe neutropenia and diarrhea (16). The prevalence of these alleles in various geographic populations have led to revisions in the product labeling of irinotecan for poor metabolizers. The U.S. Food and Drug Administration recommends an initial reduction by one dose level in *28 homozygotes (UGT1A1*28/*28) (17). The 2012 European Society for Medical Oncology (ESMO) consensus guidelines state testing for UGT1A1 polymorphisms should be considered when irinotecan is used at high doses (300–350 mg/m2) (18). However, doses in this range are rarely administered in the United States. More recent ESMO guidelines from 2016 continue to recognize that *6 and *28 polymorphisms are predictive biomarkers of irinotecan-related toxicity. This guideline further acknowledges that testing is not used in everyday practice (likely due to barriers in implementation), therefore phenotyping should be performed in patients with a suspicion of UGT1A1 deficiency as reflected by low conjugated bilirubin and in patients planning to receive doses of >180 mg/m2 (19). Other international groups acknowledge the increased toxicity risk in individuals with UGT1A1*6/*6, *28/*28, and *6/*28 genotypes and recommend testing prior to treatment (20, 21). **Table 1** describes the function and prevalence of clinically actionable *DPYD* and *UGT1A1* alleles in different populations.

**Table 1:** Description and prevalence of actionable *DPYD* and *UGT1A1* pharmacogenetic variants tested in the IMPACT-GI study.

Gene	Variant Allele			Allele Frequency^15,31^				
	* Allele	c. Nomenclature	rsID	AA	CSA	EA	EU	LAT
*DPYD*	*2A	c.1905+1G>A	rs3918290	0.003	0.005	0.000	0.008	0.001
	*8	c.703C>T	rs1801266	NR	0.0002	0.000	0.0001	0.000
	*10	c.2983G>T	rs1801268	NR	NR	NR	NR	NR
	*12	c.1156G>T	rs78060119	NR	NR	NR	NR	NR
	*13	c.1679T>G	rs55886062	0.000	0.000	0.000	0.001	0.000
	HapB3	c.1236G>A	rs56038477	0.003	0.020	0.000	0.024	0.006
		c. 1129-5923C>G	rs75017182					
		c.483+18G>A	rs56276561					
		c.557A>G	rs115232898	0.012	NR	0.000	0.0001	0.001
		c.2846A>T	rs67376798	0.003	0.001	0.000	0.004	0.002
*UGT1A1*	*6	c.211G>A	rs4148323	0.004	0.045	0.146	0.008	0.012
	*28	c.-41_-40dupTA( TA7)	rs8175347	0.373	0.414	0.148	0.316	0.400

AA, African ancestry; CSA, Central/South Asian ancestry; EA, East Asian ancestry; EU, European ancestry; LAT, Latino ancestry; N/A, Not applicable; NR, not reported.

*Refers to the standardized “star” (*) allele nomenclature.

Preemptive testing in patients receiving fluoropyrimidine- and/or irinotecan-based regimens has shown to be feasible, safe, and cost-effective in both the academic medical center and community settings (5, 12, 22–25). While these data support clinical implementation, barriers to routine testing in the clinic often include access to timely results, lack of clinician experience in interpreting actionable findings, and test costs that may be incurred by patients. The average time frame for translating research findings into practice is seventeen years (26). Implementation science facilitates the timely integration of evidence-based practice into clinical care and expands the focus from the patient level to address provider, organization, and policy level barriers in healthcare delivery. Determinant frameworks such as the Consolidated Framework for Implementation Research (CFIR) and process frameworks such as Exploration, Preparation, Implementation, Sustainment (EPIS) provide foundational approaches to introducing and evaluating a new intervention, such as PGx testing, through systematic assessment of key constructs that influence implementation and effectiveness (27, 28). Both frameworks identify inner and outer contextual factors essential to implementation; EPIS uniquely highlights sustainability, a critical component for administrators and payers interested in longer-term fiscal considerations for PGx test coverage to adopt and maintain testing at the health system level. Successful clinical implementation of PGx testing holds the potential to identify at-risk patients, personalize chemotherapy dosing, and better manage toxicity. Therefore, we sought to address barriers to *DPYD/UGT1A1* testing identified in our institution using the EPIS framework through the Implementing Pharmacogenetic Testing in Gastrointestinal Cancers (IMPACT-GI) study to maintain PGx services as a new standard of care.

### Guiding Implementation Science Framework

**Figure 1:**
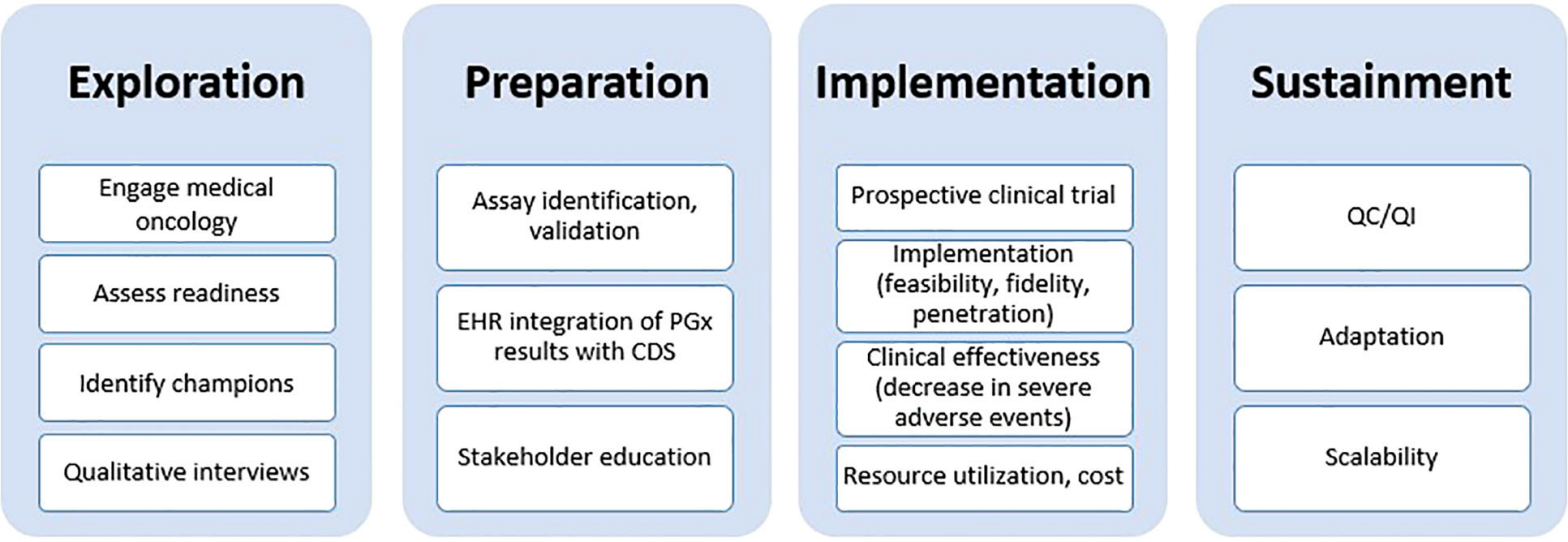
Exploration, Preparation, Implementation, Sustainment (EPIS) framework used as a guide for implementing *DPYD/UGT1A1* pharmacogenetic testing. EHR, electronic health record; PGx, pharmacogenomics; CDS, clinical decision support; QC/QI, quality control/quality improvement.

The design of this study was guided by the EPIS framework by Aarons and colleagues (**Figure 1**) (29). During the *Exploration* phase, we gauged interest in implementing *DPYD* testing from leadership within our healthcare system including the Director of the Penn Center for Precision Medicine, Director of the Cancer Center, the Chief Executive Officer, and the head of the gastrointestinal (GI) cancer service line. We evaluated various options for PGx testing such as establishing an institutional test, partnering with an academic clinical laboratory, or using a commercial laboratory. The *Preparation* phase involved an internal assessment of barriers and facilitators from the point of view of GI oncology providers curated during semi-structured qualitative interviews. We also performed a retrospective study to understand baseline rates of drug-related adverse events in patients receiving fluoropyridines and/or irinotecan. During the *Implementation* phase, we designed and refined strategies to address each of the barriers uncovered during the internal assessment; each of the strategies are described in detail in the next section. Once the study is complete, we will examine *Sustainment* of PGx testing during the transition from research testing to clinical testing.

## Methods/Design

### Qualitative Interviews

We conducted a qualitative study with our GI oncologists and oncology pharmacists to elicit impressions about current dosing practices, attitudes toward using PGx results to tailor prescribing, and perspectives on an appropriate study design for a prospective PGx trial. An interview guide informed by CFIR constructs was created to facilitate semi-structured interviews. A brief survey was distributed during the interview to collect participant demographics and quantitatively assess level of comfort in interpreting PGx results. Barriers to testing highlighted by our clinicians included a limited evidence base and burdensome workflows related to testing (e.g., lengthy turnaround time, financial concerns, EHR integration); full results of this study are published in a separate manuscript (30). Our qualitative study allowed us to identify contextual factors deemed essential to test uptake within our institution and refine strategies to address barriers during the implementation study.

### Retrospective Study

As part of our *Preparation* phase, we also conducted an institutional retrospective study to understand baseline drug-related adverse event rates in adult patients with a GI malignancy who initiated fluoropyridines and/or irinotecan over a six-month period in 2017 and 2018. We observed that approximately half of our cohort experienced at least one toxicity event (primarily related to the hematological or GI system), with 22% of patients requiring management in the emergency department or hospital. This cohort will serve as a control group for those receiving PGx testing in the prospective study. A subset of these participants have DNA available as part of the Penn Medicine Biobank and we are currently genotyping *DPYD* and *UGT1A1* variants.

### Assay Validation

Our *Exploration* phase informed plans to partner with an academic clinical laboratory to offer germline testing. Prior to the availability of our PGx panel, genotyping was typically performed as a send-out laboratory test following chemotherapy-induced toxicity to confirm enzymatic deficiency. Results took approximately four weeks to return, rendering this prolonged timeline unfeasible and impractical to guide preemptive chemotherapy dosing. During *Preparation*, we collaborated with our academic clinical laboratory to develop and validate a custom panel of twelve *DPYD* variants and two *UGT1A1* variants: *2A, *5, *6, *8, *9A, *10, *12, *13, HapB3, c.557A>G (rs115232898), c.496A>G (rs2297595), and c.2846A>T (rs67376798) for *DPYD*; and *6, and *28 for *UGT1A1*. **Table 2** provides the corresponding activity scores for each *DPYD* variant listed. The Illumina™ Infinium Global Screening Array version 3 (GSAv3.0) is used to detect variants in the *DPYD* gene and the Applied Biosystems™ fragment analysis assay is used to assess for thymine-adenine (TA) tandem repeats in *UGT1A1*, followed by Sanger sequencing confirmation for both genes. CPIC tables describing *DPYD* and *UGT1A1* allele frequencies in major ethnic groups are reviewed for updates on a quarterly basis by the laboratory (15, 31). Genotyping costs are covered by the research study.

**Table 2:** *DPYD* allele function and activity score.

DPYD * Allele/rsID	Activity Score	Allele Function
*1	1	Normal
*2A	0	None
*5	1	Normal
*6	1	Normal
*8	0	None
*9A	1	Normal
*10	0	None
*12	0	None
*13	0	None
HapB3 (rs75017182, rs56038477, rs56276561)	0.5	Decreased
rs115232898	0.5	Decreased
rs67376798	0.5	Decreased
rs2297595	1	Normal

*Refers to the standardized “star” (*) allele nomenclature.

### Integration of Pharmacogenetic Test Results Into the EHR

**Figure 2:**
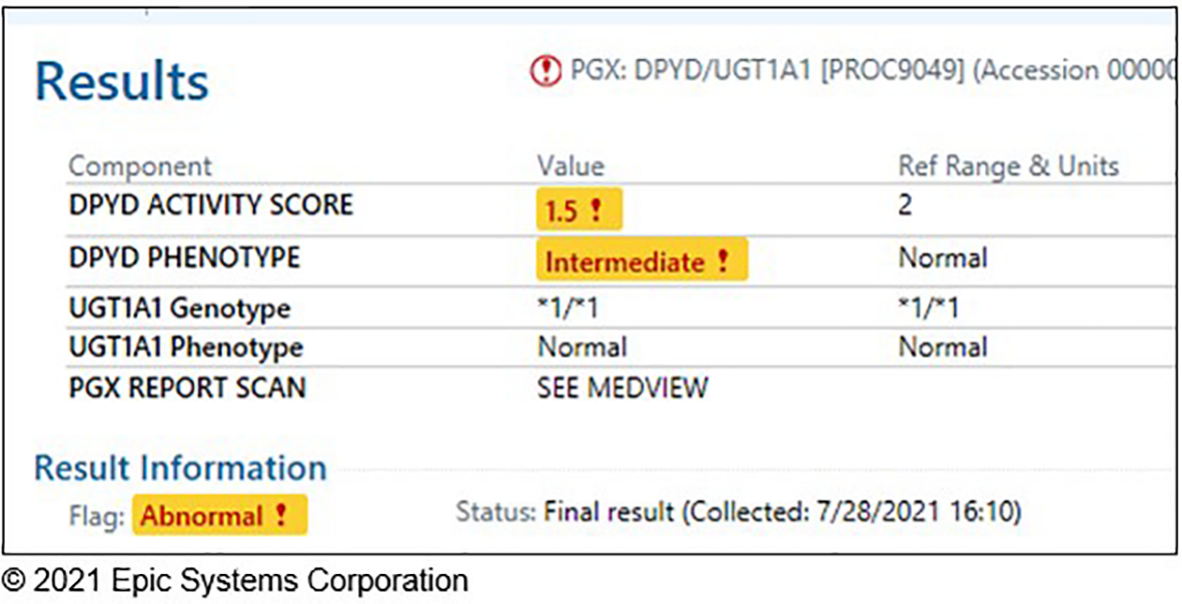
Discrete *DPYD*/*UGT1A1* genotype results in a patient chart.

Prior to study initiation, most germline genetic data were reported in unstructured portable document formats (PDF) that fragmented workflows for personalized interpretation and application. The study team worked closely with Information Services (IS) within our institution and Epic Systems Corporation (Verona, Wisconsin, USA) to customize the Genomics Module and develop the PennChart Precision Medicine tab to serve as a centralized location for pharmacogenomic information in the patient’s medical record (32). *DPYD* and *UGT1A1* genotyping results are now stored in a discrete, computable format to enable electronic searching, clinical decision support (CDS), and secondary use for research and operations. PGx results are entered as diplotypes based on PharmVar star allele definitions (e.g., *UGT1A1* *1/*28) or CPIC activity score (e.g., *DPYD* Activity Score 1.5) and mapped to the corresponding phenotype (**Figure 2**). Integration of PGx results into the EHR serves as a key strategy to ensure sustainability of the testing long-term.

**Figure 3:**
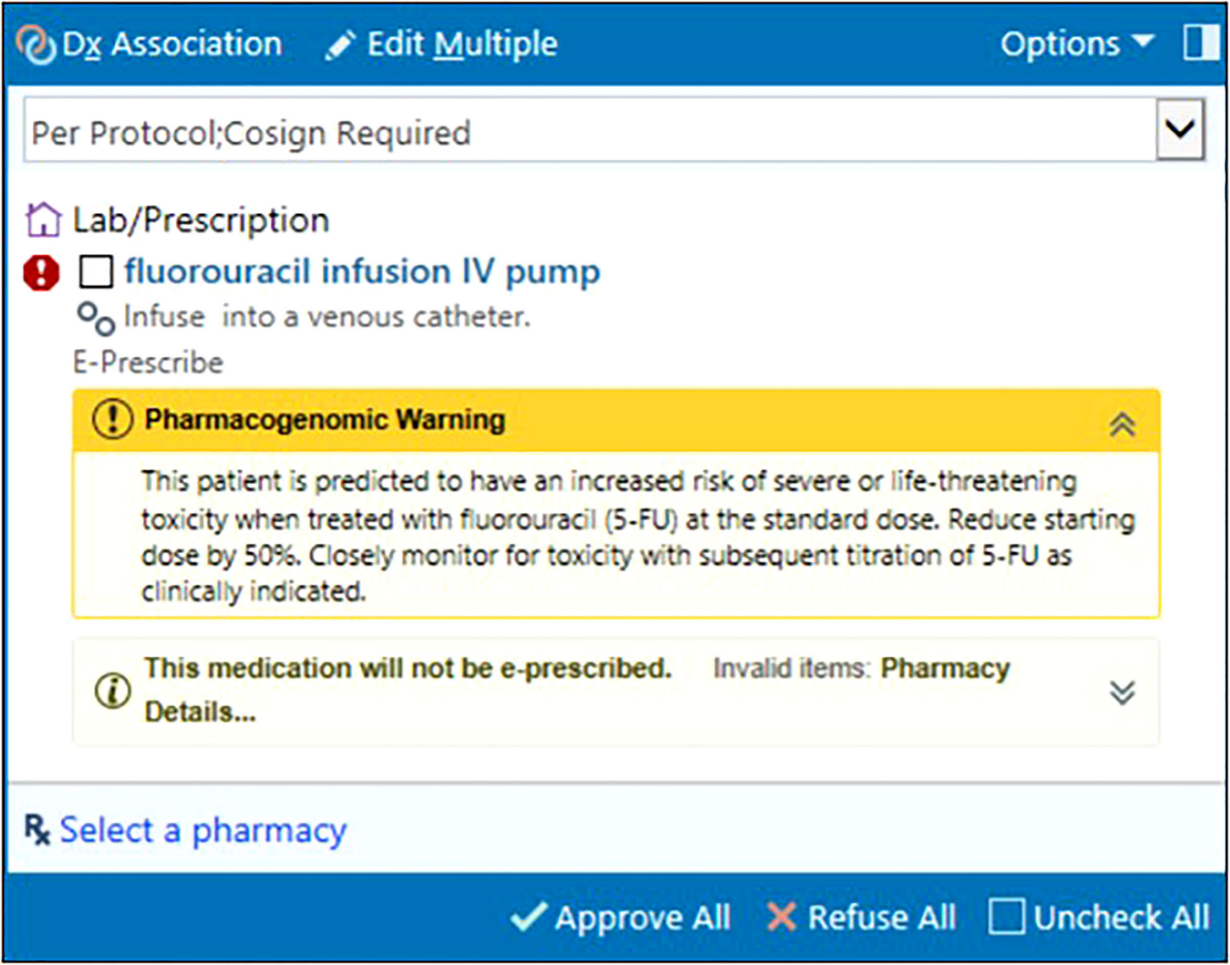
In-line warning at fluorouracil order entry informing provider of actionable DPYD results, clinical implication, and dose recommendation. © 2021 Epic Systems Corporation

Epic’s Genomic Indicators feature consists of tags added to a patient’s chart indicating PGx phenotypes based on entered results. Displayed on the Snapshot tab, clinically actionable indicators drive automated CDS in the EHR to the care team (physicians, advanced practice providers, pharmacists, and nurses). Clinicians receive a best practice alert (BPA) notifying them of results at the genotype level within the Precision Medicine tab. For patients with actionable PGx results impacting their treatment regimen, an in-line warning and pop-up alert appear in their chart at the time of chemotherapy order entry and verification. These warnings succinctly summarize the drug-gene interaction and provide a guideline-concordant dosing recommendation (**Figure 3**). Results indicating high-risk genotypes immediately impacting patients are directly communicated to the ordering physician by study personnel and pharmacy staff to prevent delays in care. **Table 3** outlines the genotype-guided CDS on *DPYD* and *UGT1A1* results integrated in our EHR system.

**Table 3:** Genotype-guided clinical decision support for *DPYD* and *UGT1A1* results.

Phenotype	Clinical Implication	Clinical Decision Support Alert Message	Reference
*DPYD* Normal Metabolizer (Activity Score 2)	Patient is predicted to have a normal risk of toxicity when treated with 5-FU or capecitabine.	None	15
*DPYD* Intermediate Metabolizer (Activity Score 1.5)	Patient is predicted to have an increased risk of severe toxicity when treated with 5-FU or capecitabine.	This patient is predicted to have an increased risk of severe or life-threatening toxicity when treated with fluorouracil or capecitabine at the standard dose. Reduce starting dose by 50%. Closely monitor for toxicity with subsequent titration of fluorouracil or capecitabine as clinically indicated.	15
*DPYD* Intermediate Metabolizer (Activity Score 1)	Patient is predicted to have an increased risk of severe toxicity when treated with 5-FU or capecitabine.	This patient is predicted to have an increased risk of severe or life-threatening toxicity when treated with fluorouracil or capecitabine at the standard dose. Reduce starting dose by 50%. Closely monitor for toxicity with subsequent titration of fluorouracil or capecitabine as clinically indicated.	15
*DPYD* Poor Metabolizer (Activity Score 0.5)	Patient is predicted to have an increased risk of severe toxicity when treated with 5-FU or capecitabine.	This patient is predicted to have an increased risk of severe or life-threatening toxicity when treated with fluorouracil or capecitabine at the standard dose. Avoid use of fluorouracil or capecitabine. If alternative agents are not considered a suitable option, administer fluorouracil or capecitabine at a strongly reduced dose (i.e. <25% of normal starting dose).	15
*DPYD* Poor metabolizer (Activity Score 0)	Patient is predicted to have an increased risk of severe toxicity when treated with 5-FU or capecitabine.	This patient is predicted to have an increased risk of life-threatening toxicity when treated with fluorouracil (5-FU) or capecitabine at the standard dose. Avoid use of 5-FU or capecitabine.	15
*UGT1A1* Normal Metabolizer (*1/*1)	Patient is predicted to have a normal risk of toxicity when treated with irinotecan.	None	17, 20, 21
*UGT1A1* Intermediate Metabolizer (*1/*28 or *1/*6)	Patient is predicted to have a normal risk of toxicity when treated with irinotecan.	None	17, 20, 21
*UGT1A1* Poor Metabolizer (*28/*28, *6/*6, or *6/*28)	Patient is predicted to have an increased risk of severe toxicity when treated with irinotecan.	The patient is predicted to have an increased risk of severe toxicity when treated with irinotecan at the standard dose. Reduce starting dose by 30%. Closely monitor for toxicity with subsequent titration of irinotecan as clinically indicated.	17, 20, 21

*Refers to the standardized “star” (*) allele nomenclature.

### Clinician Education

Our qualitative interviews highlighted the need for clinician education on PGx and the current evidence base for germline *DPYD* and *UGT1A1* screening. We used these findings to organize a Continuing Education program for oncology pharmacists and an in-service presentation for physicians one month prior to the initiation of the IMPACT-GI study. These educational initiatives focused on disseminating foundational PGx knowledge, from the role of actionable *DPYD* and *UGT1A1* variants in pharmacotherapy to current regulatory stances and evidence on the feasibility, safety, and cost utility of screening in the clinic. We also reviewed the test ordering and resulting processes in our EHR to maximize the learning experience for providers and promote its use in our oncology clinic. Ongoing PGx education is delivered through our CDS system and study newsletters. Our CDS tools provide clinicians with digestible, patient-specific result interpretations and recommendations at the point of care. Study newsletters are distributed periodically to the GI oncology team and reviewed at standing clinical research meetings to maintain clinician engagement, obtain feedback on implementation processes, and report on enrollment trends with rates of test ordering and turnaround times.

### Study Design of Implementation Trial

**Figure 4:**
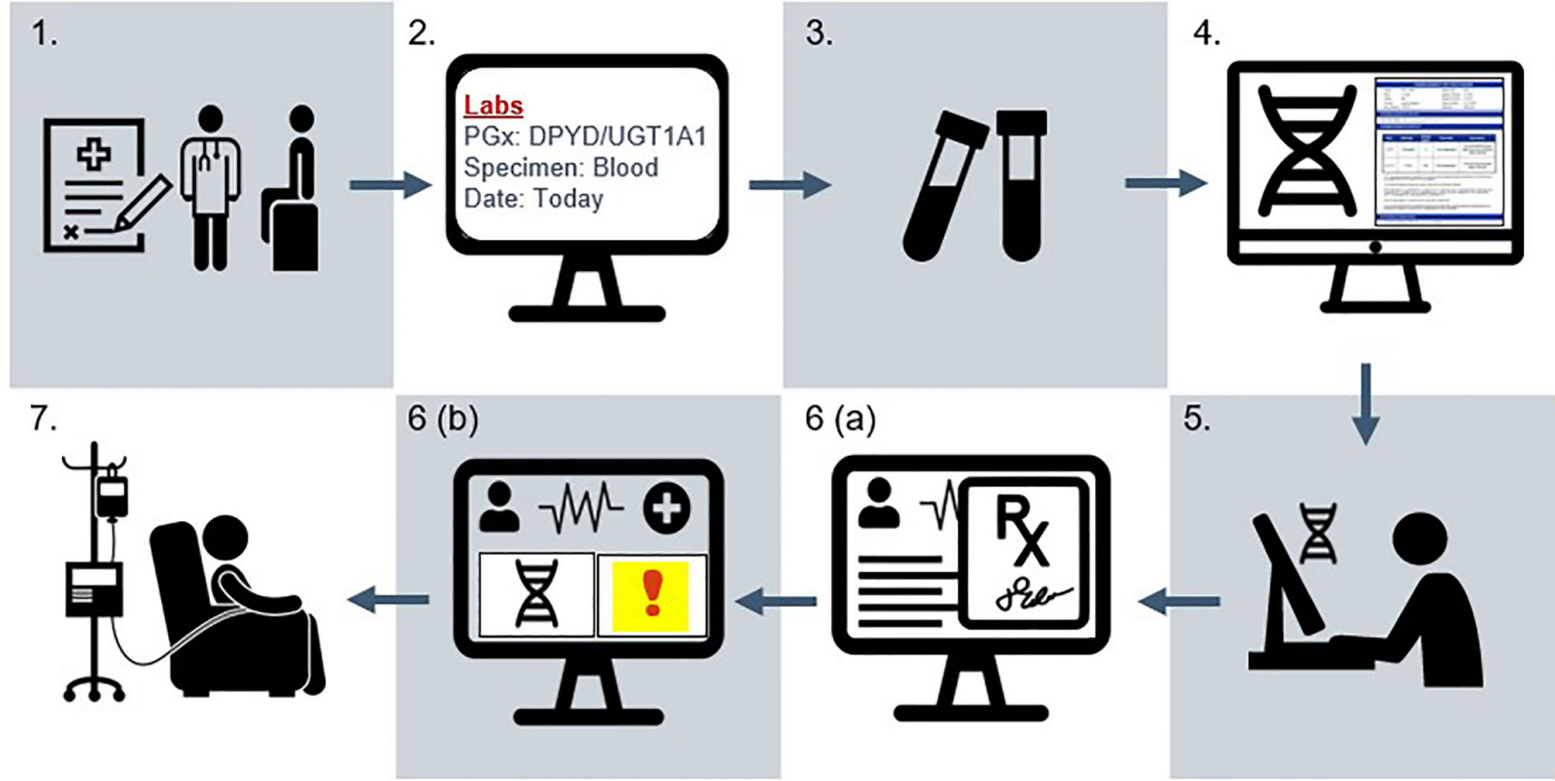
Study schema for the IMPACT-GI study. (1) Patient provides informed consent at initial evaluation (baseline) visit in the gastrointestinal oncology clinic. (2) A laboratory order for pharmacogenetic testing is placed in the EHR. (3) A specimen is collected alongside routine laboratory collections by phlebotomy. (4) The specimen is sent to an external CLIA laboratory for genotyping and report generation. (5) Pharmacogenetic results are entered by the institutional lab into the precision medicine section of the EHR as discrete result components. (6a) When the care team signs and verifies chemotherapy orders, (6b) an alert indicating increased toxicity risk appears in the patient’s chart for individuals with actionable results. Clinical decision support provides recommendations for dose adjustments. (7) Following order verification, chemotherapy is prepared and dispensed to the patient in the oncology infusion suite. Fidelity is demonstrated when the prescriber adjusts dosing according to the patient’s genotype. Adverse event data is collected for the first six cycles of treatment. Patients complete a symptom questionnaire at each of these cycles and a one-time survey assessing attitudes towards pharmacogenetic testing after receiving at least two cycles. Tumor outcomes (progression-free survival, overall survival) are assessed at approximately six months following treatment initiation.

This is a pragmatic, non-randomized, open-label, multi-site trial performed within the University of Pennsylvania Health System (UPHS) (Philadelphia, Pennsylvania, USA). UPHS is an academic health system of six acute-care hospitals with approximately 3000 beds and over five million annual outpatient visits. Eligible participants are recruited at two oncology clinics in Philadelphia and an outpatient cancer center in Lancaster, Pennsylvania. **Figure 4** presents the study workflow.

### Study Objectives

The primary objective of the IMPACT-GI study is to assess the feasibility of introducing *DPYD* and *UGT1A1* pharmacogenetic testing to guide initial fluoropyrimidine and irinotecan dosing in patients with GI malignancies. The secondary objectives are to evaluate the clinical effectiveness of the PGx testing service using clinical data, patient-reported outcome (PRO), and quality of life (QoL) measures. We hypothesize that the availability of a rapid turnaround PGx test with specific dosing recommendations will increase PGx test utilization to inform pharmacotherapy decisions and improve patient safety outcomes through our prospective clinical implementation study.

### Study Outcomes

Primary endpoints for this implementation study are to determine (1): feasibility, defined by the proportion of PGx test results returned prior to the first dose of chemotherapy (2); fidelity, or the proportion of dose modifications made in agreement with genotype-guided dosing recommendations; and (3) penetrance, characterized by the rate of testing among eligible patients at our study sites.

Feasibility and fidelity data will be prospectively collected on a continual basis by examining the timestamp on test orders and pharmacy records. Penetrance will be determined by examining clinic schedules and study screening logs for eligible patients.

Exploratory outcomes for include (1): the proportion of patients experiencing ≥Grade 3 toxicity according to NCI Common Terminology Criteria for Adverse Events (CTCAE) version 5.0 (33) over the first six cycles of chemotherapy (or fewer if planned); 2) the relative dose intensity of fluoropyrimidine and irinotecan dosing over the first six cycles for comparison with the historical control cohort (3); PRO and QoL responses during the first six cycles of chemotherapy (34, 35); and (4) participant knowledge and attitudes towards PGx testing as determined by a patient survey. Additional exploratory endpoints include (1): the incidence of ≥Grade 3 toxicity by ancestry (2); minor allele frequencies for *DPYD* and *UGT1A1* reported by ancestry (3); survival analysis (progression-free survival, PFS and overall survival, OS) (4); medical service utilization and costs; and (5) assessment of exploratory biomarkers with fluoropyrimidine-associated toxicity.

The study team will review participants’ medical records to collect demographic and clinical data at baseline, the first six cycles of chemotherapy (or fewer if planned), and survival data approximately six months from treatment initiation. PRO and QoL questionnaires will be distributed electronically *via* Research Electronic Data Capture (REDCap) (36) or by paper in clinic to better understand any treatment-related symptoms from the participant’s perspective with each cycle. Information readily available in the EHR, such as clinical progress notes, telephone encounters, and patient portal messages, will be corroborated with available PRO and QoL responses to grade adverse events.

### Study Participants

Patients aged 18 years or older with a pathologically confirmed GI malignancy for which treatment with a fluoropyrimidine and/or irinotecan is indicated and a life expectancy of at least six months are eligible. Initially inclusion was restricted to patients with an ECOG performance status of 0, 1, or 2, but the protocol was amended in December of 2021 to no longer restrict testing based on functional status. Participants must be able and willing to provide informed consent and undergo blood sampling for genotyping and comply with study procedures.

Exclusion criteria include (1): known *DPYD* and *UGT1A1* genotype status (2); unacceptable laboratory values, including (a) hepatic dysfunction, as defined by serum bilirubin ≥1.5 x upper limit of normal (ULN), alanine aminotransferase (ALT), and aspartate aminotransferase (AST) ≥2.5 x ULN, or in case of liver metastases ALT and AST≥5 x ULN, (b) renal dysfunction as defined by serum creatinine ≥1.5 x ULN, or creatinine clearance <60 ml/min (by Cockcroft-Gault Equation), or (c) absolute neutrophil count of <1.5 x 10^9^/L or platelet count of <100 x 10^9^/L (3); women who are pregnant or breast feeding, or subjects who refuse to use reliable contraceptive methods throughout the study; and (4) treating physician does not want the subject to participate. Initially prior treatment with 5-fluorouracil or capecitabine was an exclusion, but this was later amended in December 2021 to enroll patients who had received these agents in the past.

The duration of patient recruitment is 18 months. The follow-up period for enrolled participants is six months from the first dose of chemotherapy. Participants are free to withdraw from participation in the study at any time without stating any reason nor affecting their medical care.

### Sample Size

We plan to enroll 300 participants at the three sites. This number is a convenience sample based on known patient volume from institutional cancer registry data, estimating the number of patients that will be eligible for testing. The first 116 participants were enrolled prior to the study amendment.

### Study Procedures

#### Recruitment

All recruitment is conducted at three Penn Medicine cancer clinics. Clinic schedules are screened by clinical research personnel for patients diagnosed with a GI tumor being evaluated for treatment. Prior to the scheduled initial office visit, the research coordinator confirms eligibility with the treating oncologist. The oncologist or research coordinator then discusses the study with the patient during this visit and obtains consent for study participation.

Following enrollment, an order is placed within the patient’s medical record so that a blood sample for DNA genotyping is obtained alongside routine laboratory orders by clinic phlebotomists. High-throughput genotyping, interpretation, and report generation is carried out in a College of American Pathologists (CAP)-accredited and Clinical Laboratory Improvement Amendments (CLIA)-certified laboratory at the Center for Applied Genomics at the Children’s Hospital of Philadelphia (Philadelphia, PA, USA). The anticipated test turnaround time is ten business days.

#### Study Assessments

• Baseline (collected at time of enrollment):

o Demographics: age, gender, race/ethnicity, insurance status, work status, and contact information for electronic PRO, QoL, and survey responses

o Cancer history: GI tumor type, stage, history or planned surgical resection and/or radiation therapy, previous lines of therapy, and treatment intent with prescribed regimen

o Laboratory assessments: vital signs (height, weight, heart rate, blood pressure), routinely performed laboratory tests: complete metabolic panel (CMP), complete blood count (CBC), and Eastern Cooperative Oncology Group (ECOG) performance status

o Concomitant medications

• Ongoing (collected during chemotherapy cycles 1-6, or fewer if planned):

o Laboratory assessments: vital signs, CMP, CBC, and ECOG performance status

o Concomitant medications (changes from baseline or previous cycle)

o PRO and QoL responses (obtained by paper or electronically at each subsequent visit to reflect symptoms experienced in the previous cycle)

o Toxicity events and management (including treatment location as outpatient or emergency department/hospitalization, and changes to prescribed regimen)

• End of study (collected at six months from treatment initiation):

o Subject status (completion of planned toxicity assessments, study withdrawal, or death)

o Laboratory assessments: vital signs, CMP, CBC, and ECOG performance status

o Concomitant medications (changes from baseline or previous cycle)

o Oncologic outcomes: PFS and OS based on available clinical data

#### Patient Survey

Participant-reported knowledge and attitudes towards PGx testing will be captured by a REDCap survey instrument (**Supplementary Data Sheet 1**). To support standardization among participants enrolled at different time points, the survey is disseminated to genotyped participants who have received at least two cycles of treatment. A paper-based version is available for patients who wish to complete the survey in clinic or may lack internet access. Participants will be compensated with a $25 gift card for survey completion. Likert scale responses will be compared by sex, race/ethnicity, tumor type, and socioeconomic status using linear regression.

#### Statistical Plan

Primary implementation endpoints will be analyzed using descriptive statistics. The mean, standard deviation (SD), median, interquartile range, range, counts, and percentage will be used to describe and compare baseline characteristics between the prospectively genotyped group and the historical control cohort. Student’s t-test or rank sum test will be employed for continuous variables and Fisher’s exact test for categorical variables.

While our primary study outcomes are focused on evaluating implementation, our exploratory measures of clinical effectiveness will employ a multivariable regression model with adjustment for covariates (e.g. age, sex, tumor type, treatment regimen, ECOG performance status) to compare the proportion of ≥Grade 3 toxicities in variant carriers who received genotype-adjusted dosing in the prospective IMPACT-GI cohort to variant carriers who received standard chemotherapy dosing in the historical control group. Subgroup analyses will be performed by tumor type. PRO and QoL responses as assessed on a Likert scale will be reported as means (SD).

### Concurrent Process Evaluation

To further delineate the barriers that arose during the implementation study, we will elicit feedback from central users involved in the implementation process. We will perform one-on-one semi-structured key informant interviews at the end of the study with the following individuals: oncology physicians, advanced practice providers, pharmacists, research and clinical support staff, Information Services staff, and laboratory staff. We will also examine perspectives from health system administrators and local payers. These efforts will also be essential in evaluating, refining, and sustaining future PGx efforts in our institution.

### Present Status

The first patient was enrolled in March 2021. As of January 2022, 116 participants have been enrolled across the three sites. Baseline characteristics of those patients are shown in **Table 4**. Recruitment of the last patient is expected in August 2022.

**Table 4:** Baseline characteristics of the first 116 participants.

	n=116
**Age, years (mean + SD)**	61 + 13.2
**Sex, female, n (%)**	57 (49)
**Ancestry/Ethnicity, n (%)**	
White	77 (66.4)
Black	25 (21.6)
Hispanic/Latino	7 (6.0)
East Asian	5 (4.3)
Other	2 (1.7)
**Tumor type, n (%)**	
Colorectal	51 (44)
Pancreas	33 (28.4)
Appendix	9 (7.7)
Gastric	5 (4.3)
Small intestine	5 (4.3)
Esophageal	4 (3.4)
Other	9 (7.7)
**Treatment regimen, n (%)**	
FOLFOX-based	47 (40.5)
Capecitabine-based	35 (30.2)
FOLFIRI-based	27 (23.3)
Other	7 (6)

## Discussion

Precision medicine initiatives have accelerated the translation of genomics research into clinical practice and continue to gain traction in health systems. It is anticipated that clinical adoption of PGx testing will become more ubiquitous with growing stakeholder interest and increasing test coverage policies by major payers (37–39). Implementation science frameworks such as EPIS provide a roadmap for implementation and can facilitate the adoption of PGx testing into routine clinical care. We established implementation in one service line (GI oncology) to build necessary clinical operations to deliver PGx testing. Conducting qualitative interviews during the Exploration phase were vital in identifying barriers to implementation and at the same time provided an opportunity to engage and educate key personnel about the intervention (30).

While randomized controlled trials (RCTs) remain the gold standard for medical practice, alternative pragmatic methods should be considered for contributing to real-world PGx evidence and supporting its clinical use (40–42). Given that many actionable PGx markers occur at low frequencies in the population, it is not always feasible to conduct a RCT and demonstrate effectiveness with sufficient power. During our implementation planning process, many oncologists in our institution expressed ethical concerns with a randomized trial design, fearing that a *DPYD* or *UGT1A1* carrier may receive chemotherapy at standard dosing and thereby be exposed to an increased risk of toxicity. Bearing these contextual factors in mind, we pursued a non-randomized, open-label approach to our prospective study with the goal of establishing the PGx test as part of routine care and removing barriers to its use. Additionally, well-powered effectiveness trials have already been performed showing that prospective *DPYD* testing reduces severe toxicity; our study design is similar to other implementation science trials with the primary goal of demonstrating feasibility and fidelity in individualizing chemotherapy dosing (12, 43). To our knowledge, this is the first study prospectively evaluating additional *DPYD* and *UGT1A1* variants such as *DPYD* c.557A>G and *UGT1A1* *6, which are observed more frequently in populations of non-European ancestry and reflect the diversity of patient populations receiving care in our health system. It should be further acknowledged that individual germline genetic variations play one role in the clinical outcomes of chemotherapy treatment, other factors such as gender, age, weight, lifestyle habits, performance status, organ dysfunction and concomitant medications must also be taken into consideration for determining treatment plans. An interdisciplinary clinical team that includes a pharmacist is crucial for evaluating drug-drug interactions and drug-drug-gene interactions from concomitant medications and PGx profiles for optimal dosing decisions, particularly in an aging cancer population where polypharmacy is highly prevalent (44, 45).

Following study conclusion, we intend to scale this testing to patients with other tumor types (e.g., breast, head and neck) considering fluoropyrimidine therapy, along with plans to offer testing of additional PGx variants to guide prescribing of supportive care medications administered during chemotherapy (e.g., anti-emetics, analgesics). Local laboratory partnerships, EHR infrastructure build, and new regional test coverage for PGx testing has laid the groundwork for future test panels in our health system. Implementation science framework will continue to shape implementation strategies across our multi-level health system to bridge gaps between the available evidence and delivery of care.

## Conclusion

Fluoropyrimidines and irinotecan remain commonly prescribed chemotherapy agents for GI malignancies. Screening for germline *DPYD* and *UGT1A1* variants to tailor chemotherapy dosing to each patient’s genetic profile can help identify those at highest risk for toxicity to improve patient outcomes while achieving favorable risk/benefit ratios of treatment tolerability and efficacy. This study leverages implementation science frameworks to evaluate the implementation of *DPYD* and *UGT1A1* testing, while developing infrastructure for genomic medicine in our cancer centers to ensure sustainability of PGx testing as standard of care.

## Data Availability Statement

The raw data supporting the conclusions of this article will be made available by the authors, without undue reservation.

## Ethics Statement

The studies involving human participants were reviewed and approved by both the institutional review board (IRB) and the Abramson Cancer Center Clinical Trials Scientific Review and Monitoring Committee (CTSRMC) at the University of Pennsylvania as of 17 December 2020. It is registered at ClinicalTrials.gov (NCT04736472). The patients/participants provided their written informed consent to participate in this study.

## Author Contributions

All authors listed have made a substantial, direct, and intellectual contribution to the work and approved it for publication.

## Funding

This study is funded by the Penn Center for Precision Medicine. LV is supported by the National Center for Advancing Translational Sciences of the National Institutes of Health TL1TR001880. KL-M is supported by T32HG009495. ST is supported by K23HL143161.

## Conflict of Interest

The authors declare that the research was conducted in the absence of any commercial or financial relationships that could be construed as a potential conflict of interest.

## Publisher’s Note

All claims expressed in this article are solely those of the authors and do not necessarily represent those of their affiliated organizations, or those of the publisher, the editors and the reviewers. Any product that may be evaluated in this article, or claim that may be made by its manufacturer, is not guaranteed or endorsed by the publisher.

## Supplementary Material

Supplementary Data Sheet 1 Perceptions of Pharmacogenetic Testing in Patients with GI Cancers (patient survey).
