# Supplementary material for: Implementing Pharmacogenetic Testing in Gastrointestinal Cancers (IMPACT-GI): Study Protocol for a Pragmatic Implementation Trial for Establishing DPYD and UGT1A1 Screening to Guide Chemotherapy Dosing
Source: Front Oncol. 2022 Jul 5;12:859846. doi: 10.3389/fonc.2022.859846 (PMC9295185; doi:10.3389/fonc.2022.859846)
Supplement: Supplementary Data Sheet 1 — Perceptions of Pharmacogenetic Testing in Patients with GI Cancers (patient survey). [file DataSheet_1.pdf]

Perceptions of Pharmacogenetic Testing in Patients with GI Cancers

Please complete the survey below. The survey should take about 15 minutes to complete.

Thank you!

---

1. First name

____

2. Last name

____

3. Please enter your age:

____

4. What is the highest degree or level of school you have completed?

- ◯ Less than high school
- ◯ High school graduate (or GED)
- ◯ Some college/university
- ◯ College/university graduate
- ◯ Post college/university degree
- ◯ Choose not to respond

5. What is your marital status?

- ◯ Married
- ◯ Living as married/Domestic Partnership
- ◯ Divorced
- ◯ Widowed
- ◯ Separated
- ◯ Never been married

6. What is your combined annual income (total pre-tax income from all sources earned in the past year)?

- ◯ $0-$9,999
- ◯ $10,000-$14,999
- ◯ $15,000-$19,999
- ◯ $20,000-$34,999
- ◯ $35,000-$49,999
- ◯ $50,000-$74,999
- ◯ $75,000-$99,999
- ◯ $100,000-$199,999
- ◯ $200,000 or more
- ◯ Choose not to respond

7. How confident are you in filling out forms in a doctor's office by yourself?

- ◯ Extremely
- ◯ Quite a bit
- ◯ Somewhat
- ◯ A little bit
- ◯ Not at all

8. Over the past month how would you describe your health?

◯ Poor ◯ Fair ◯ Good
◯ Very Good ◯ Excellent

9. Have you ever had a bad reaction from chemotherapy or immunotherapy? (select all that apply)	☐ Yes, Chemotherapy<br>☐ Yes, Immunotherapy<br>☐ No, I have NEVER had a bad reaction from either chemotherapy or immunotherapy<br>☐ I am unsure
9b. How would you describe your reaction to chemotherapy or immunotherapy?	☐ I have had a bad reaction from chemotherapy or immunotherapy that led to me STOPPING it.<br>☐ I have had a bad reaction from chemotherapy or immunotherapy that led to a DOSE REDUCTION.<br>☐ I have had a bad reaction from chemotherapy or immunotherapy that did not lead to any changes in my treatment.<br>☐ I am unsure
10. Apart from chemotherapy or immunotherapy, have you ever had a bad reaction from a medicine?	☐ Yes, I have had a bad reaction from a medicine that led me to STOPPING it<br>☐ Yes, I have had a bad reaction from a medicine that led to a DOSE REDUCTION<br>☐ No, I have NEVER had a bad reaction from a medicine<br>☐ I am unsure
11. Apart from chemotherapy or immunotherapy, have you ever been hospitalized from a bad reaction to a medicine?	○ Yes, I have been hospitalized due to a bad reaction to a medicine<br>○ No, I have NEVER been hospitalized due to a bad reaction to a medicine<br>○ I am unsure
11b. If you answered yes to either question 10 or 11, which medication caused the reaction?	________________________________<br>(If you do not remember, leave field blank)
12. Have you, or anyone in your family, ever had a genetic test to predict or diagnose a disease or condition?	○ Yes<br>○ No<br>○ I am unsure
13. Have you, or anyone in your family, ever had a pharmacogenetic test for the purpose of medication management?	○ Yes<br>○ No<br>○ I am unsure

The following section includes questions about the role of genetics in health and disease. When we refer to a variant, we are referring to a difference in your DNA, which may or may not impact your health.

Please check a circle to indicate how much you agree or disagree with each statement.

	Strongly Disagree	Disagree	Neither Agree nor Disagree	Agree	Strongly Agree
14. A health care provider can always tell a person their exact chance of developing a disease based on their results from genetic testing.	○	○	○	○	○
15. If a person has a variant in a gene that increases their risk of a disease, they may not develop that disease.	○	○	○	○	○
16. Genetic testing may find variants in a person's genes that could determine how they respond to certain medicines.	○	○	○	○	○
17. A person's health habits, like diet and exercise, can affect whether or not their genes can cause disease.	○	○	○	○	○
18. Cancer can be caused by genes inherited from parents.	○	○	○	○	○
19. Cancer can be caused by changes in genes over a person's lifetime.	○	○	○	○	○
20. I am confident in my ability to understand information about genetics.	○	○	○	○	○

A pharmacogenetic test is a type of genetic test that could allow doctors to choose the right medication that will work for a patient or avoid using certain medications that may cause side effects.

To what extent do you agree or disagree with the following statements?

	Strongly Disagree	Disagree	Neither Agree nor Disagree	Agree	Strongly Agree
21. I would agree to a pharmacogenetic test, if it could predict whether a medication would work for my condition.	○	○	○	○	○
22. I would agree to a pharmacogenetic test, if it could predict the correct dose of the medication that I needed.	○	○	○	○	○
23. I would agree to a pharmacogenetic test, if it could predict whether I would have a mild side effect to a drug.	○	○	○	○	○
24. I would agree to a pharmacogenetic test, if it could predict whether I would have a serious side effect to a drug.	○	○	○	○	○
25. I would agree to a pharmacogenetic test, if it could explain a family history of medication side effects or nonresponse.	○	○	○	○	○
26. I would agree to a pharmacogenetic test, if my healthcare provider recommended it.	○	○	○	○	○
27. It is important that my healthcare provider tells me about pharmacogenetic tests before any of them are done.	○	○	○	○	○
28. If a pharmacogenetic test will be performed with my usual blood work, it is important that my healthcare provider obtains my consent for the test.	○	○	○	○	○

A pharmacogenetic test is a type of genetic test that could allow doctors to choose the right medication that will work for a patient or avoid using certain medications that may cause side effects.

To what extent do you agree or disagree with the following statements?

	Strongly Disagree	Disagree	Neither Agree nor Disagree	Agree	Strongly Agree
29. Prior to undergoing a pharmacogenetic test, I would want to know how the test results could affect my treatment.	○	○	○	○	○
30. Prior to undergoing a pharmacogenetic test, I would be concerned whether a possible result would make it more difficult to find an effective treatment.	○	○	○	○	○
31. Prior to undergoing a pharmacogenetic test, I would be concerned whether a possible result would delay my treatment.	○	○	○	○	○
32. Prior to undergoing a pharmacogenetic test, I would be concerned whether a possible result would limit the treatment options made available to me.	○	○	○	○	○
33. If I had to pay for a pharmacogenetic test myself, the financial cost of the test would be of concern to me.	○	○	○	○	○
34. If I had a pharmacogenetic test, I would be concerned that insurance companies may use the pharmacogenetic test results to deny healthcare coverage.	○	○	○	○	○
35. If I had a pharmacogenetic test, I would be concerned about the effect of the pharmacogenetic test results on my employment opportunities.	○	○	○	○	○
